# Evaluation of the lncRNA-miRNA-mRNA ceRNA network in lungs of miR-147 ^−/−^ mice

**DOI:** 10.3389/fphar.2024.1335374

**Published:** 2024-03-06

**Authors:** Nan Zhang, Gui-Yuan Song, Qing-Hua Yu, Xin-Ming Fan, Wen-Shuo Zhang, Yong-Jian Hu, Tian-Zhu Chao, Yao-Yao Wu, Shu-Yan Duan, Fei Wang, Rui-Peng Du, Ping Xu

**Affiliations:** ^1^ Laboratory of Radiation-Induced Diseases and Molecule-Targeted Drugs, School of Food and Biomedicine, Zaozhuang University, Zaozhuang, Shandong, China; ^2^ School of Pharmacy, Weifang Medical University, Weifang, Shandong, China; ^3^ School of Public Health, Weifang Medical University, Weifang, Shandong, China; ^4^ Department of Radiotherapy, Zaozhuang Municipal Hospital, Zaozhuang, Shandong, China; ^5^ Henan Key Laboratory of Medical Tissue Regeneration, Xinxiang Medical University, Xinxiang, Henan, China

**Keywords:** miRNA-147, KCNH6, ceRNA, lncRNA-miRNA-mRNA network, cancer

## Abstract

**Background:** Previous studies have documented important roles for microRNA-147 (miR-147) in inflammation, radiation-induced injury, cancer, and a range of other diseases. Murine lungs exhibit high levels of miRNA, mRNA, and lncRNA expression. However, very little research to date has focused on the lncRNA-miRNA-mRNA competing endogenous RNA (ceRNA) networks associated with miR-147, and the regulation of lncRNAs and miRNAs in this setting remains poorly understood.

**Methods:** After establishing a miR-147^−/−^ model mouse, samples of lung tissue were harvested for RNA-sequencing, and differentially expressed lncRNAs, miRNAs, and mRNAs were identified. The miRNA targets of these lncRNAs and the identified miRNAs were first overlapped to facilitate the prediction of target mRNAs, with analyses then examining the overlap between these targets and mRNAs that were differentially expressed. Then, these target mRNAs were subjected to pathway enrichment analyses. These results were ultimately used to establish a miR-147-related ceRNA network.

**Results:** Relative to wild-type mice, the lungs of miR-147^−/−^ mice exhibited 91, 43, and 71 significantly upregulated lncRNAs, miRNAs, and mRNAs, respectively, together with 114, 31, and 156 that were significantly downregulated. The lncRNA-miRNA-mRNA network established based on these results led to the identification of Kcnh6 as a differentially expressed hub gene candidate and enabled the identification of a range of regulatory relationships. KEGG pathway enrichment showed that the mRNA targets of differentially expressed lncRNAs and miRNAs in the mice were associated with tumor-related signaling, endometrial cancer, bladder cancer, and ErbB signaling.

**Conclusion:** These results suggest that the identified ceRNA network in miR-147^−/−^ mice shapes tumor-associated signaling activity, with miR-147 potentially regulating various lncRNAs and miRNAs through Kcnh6, ultimately influencing tumorigenesis. Future studies of the lncRNA, miRNA, and mRNA regulatory targets shown to be associated with miR-147 in the present study may ultimately lead to the identification of novel clinically relevant targets through which miR-147 shapes the pathogenesis of cancer and other diseases.

## Introduction

Non-coding RNAs (ncRNAs) have attracted much interest in recent years owing to advances in sequencing and bioinformatics techniques, contributing to a growing understanding of the biological importance and functions of microRNAs (miRNAs) and long ncRNAs (lncRNAs). As 21-24 nucleotide ncRNAs, miRNAs interact with the 3′-untranslated region (UTR) of specific mRNA targets and promoting degradation or reduced translation (Sandquist and Wong, 2014; Afonso-Grunz and Muller, 2015). Conversely, lncRNAs are over 200 nucleotides long and are capable of functioning as miRNA sponges that sequester these ncRNAs to interfere with their function, in addition to interacting with certain mRNAs(Mattick and Rinn, 2015). Dysregulated miRNA and lncRNA expression has increasingly been tied to the pathogenesis of inflammation, Alzheimer’s disease, cancer, and a range of other diseases (Cheng, 2015). The competing endogenous RNA (ceRNA) concept was first suggested by Salmena et al., in 2011, and posits that miRNA response elements (MREs) are present within mRNAs, lncRNAS, and other types of ncRNAs, thereby enabling ncRNAS to interfere with miRNA functionality by outcompeting mRNAs for sequence-specific mRNA binding (Salmena et al., 2011). While biologically interesting, research focused on validating this ceRNA hypothesis has been relatively limited to date.

First identified as a tumor suppressor candidate expressed in murine macrophages and splenic tissue, microRNA-147 (miR-147) has more recently been shown to regulate inflammation and macrophage functionality by targeting Toll-like receptor 4 (TLR4) in addition to shaping the incidence of inflammatory bowel disease and coronary atherosclerosis (Lagos-Quintana et al., 2002; Liu et al., 2009). The upregulation of this miRNA has been reported in squamous cell carcinomas of the tongue and esophagus, as well as gastric cancer, whereas its downregulation has been observed in colon cancer (Wong et al., 2008; Yao et al., 2009; Shen et al., 2018; Tang et al., 2018; Ning et al., 2019). At a functional level, miR-147 can reportedly target and suppress brain-derived neurotrophic factor (BDNF) expression to suppress non-small cell lung cancer (NSCLC) cell migratory, proliferative, and invasive activity (Li et al., 2020). In contrast, the upregulation of miR-147 in small-cell lung cancer has been tied to enhanced chemoresistance (Zhang et al., 2019). Prior research conducted by our group suggests that miR-147a is capable of regulating AKT and 3-phosphoinositide-dependent protein kinase 1 (PDPK1) transcription in the context of radiotherapy through its ability to bind the PDPK1 3′-UTR. By inhibiting miR-147 and stimulating nuclear paraspeckle assembly transcript 1 (NEAT1) to promote PDPK1 upregulation, trixrutin was further found to enhance radioprotection. Overall, these past data highlight the varied and complex roles that miR-147 played in various biological contexts. However, there has been little research to date focused on establishing a miR-147-related ceRNA network composed of lncRNAs, miRNAs, and mRNAs. As such, lung samples from miR-147^−/−^ mice were herein used for transcriptomic sequencing and bioinformatics analyses aimed at clarifying the ceRNA networks associated with this miRNA. Together, these data will provide a foundation for research centered on clarifying the role that miR-147 plays in the pathogenesis of cancer and other diseases.

## Material method

### Mouse models

C57BL/6N and miR-147^−/−^ mice (4 weeks old) were acquired from Beijing Wei-tong-li-Hua Experimental Animal Technology Co. And the Chinese-French Immunoregulatory Genes Laboratory of Xinxiang Medical University, respectively. The deletion of miR-147 in these mice and associated genotypic changes were reported previously (Song et al., 2023). Mice were individually housed under controlled conditions (22°C, 55% humidity, 12 h light/dark cycle). Animals were euthanized prior to lung tissue harvesting. The Science and Technology Ethics Committee of Zaozhuang University approved all animal protocols.

### RNA-sequencing

TRIzol (Invitrogen, MA, United States of America) was for RNA extraction from lung tissue samples. To construct a lncRNA library, Caliper Labchip GX and a DNA 1K Reagent Kit were used to assess RNA quality, using 500 ng of murine RNA. After rRNA removal with an RNase H kit, RNA fragmentation was performed, and RNAs were reverse transcribed for first-strand cDNA synthesis, followed by dUTP-based second-strand cDNA synthesis. Following end repair, 1 A nucleotide was ligated to the 3′ end of the cDNA segment, followed by PCR amplification with the following settings: 95°C for 3 min; 14 cycles of 95°C for 30 s, 56°C for 30 s, 72°C for 1 min; 72°C for 5 min. For miRNA library construction, an Agilent 2,100 Bioanalyzer and an Agilent DNA 1000 Kit were used for assessing RNA quality. Then, 5,000 ng samples of RNA were used to purify RNAs 18-30 nucleotides in length via polyacrylamide gel electrophoresis. These transcripts were then connected at the 3′ and 5’ ends, and reverse transcription and PCR amplification were performed, collecting PCR products via polyacrylamide gel electrophoresis and storing them in EB buffer. PCR products from these libraries were denatured into single-stranded forms and cyclized to yield single-stranded circular DNAs, which were then used for rolling cycle amplification, generating a DNA nanoball (DNB) containing multiple DNA copies. DNBs of sufficient quality were then loaded into patterned nanoarrays with a high-intensity DNA nanochip technique, followed by sequencing performed through combinatorial probe-anchor synthesis.

### lncRNA-miRNA-mRNA network establishment and analysis

Sequencing data were initially filtered using SOAPnuke, and clean reads were stored in the FASTQ format, after which the Dr. Tom Multi-omics Data Mining System (https://biosys.bgi.com) was used for data analyses and data mining. Filtered clean reads were mapped with Bowtie2 to reference genomes and sRNA databases, after which RNAhybrid, miRanda, and TargetScan were used for predicting gene targets of lncRNAs and miRNAs, enabling the visualization of lncRNA-miRNA-mRNA ceRNA networks. RSEM (v1.3.1) was used to evaluate relative gene expression, and differentially expressed (DE) genes were identified with DESeq2 (v1.4.5) based on Q-values ≤0.05. GO and KEGG enrichments of DE mRNAs were analyzed with Phyper based upon hypergeometric distributions. Strong Q values (Q ≤ 0.05) were used to correct networks and significantly enriched terms.

### Cell culture

BeNa Culture Collection of China (BNCC364345) and Cellverse Bioscience Technology Co., Ltd. (iCell-h017) were the sources of MLG2908 and HFL-1 cells, which were cultured with DMEM (Hyclone) or Ham’s F-12K(PM150910) medium that had been supplemented using 10% fetal bovine serum (FBS; Gibco), penicillin (100 U/mL), and streptomycin (100 μg/mL, Hyclone), with all culture being performed at 37°C in a 5% CO_2_ incubator.

### Immunofluorescence

Cells in confocal dishes were fixed using 4% paraformaldehyde, permeabilized for 10 min using 0.2% Triton X-100, rinsed thrice with PBS, blocked for 30 min with 1% BSA, and probed overnight with anti-Kcnh6 (Santa Cruz, sc-135959, 1:200) at 4°C. Cells were again washed, probed for 2 h with a secondary antibody (Beyotime, China), subjected to nuclear counterstaining for 10 min using DAPI (Beyotime, China), washed with PBS, and imaged via fluorescence microscopy.

### Statistical analysis

Data are means ± standard deviation (SD) and were compared with GraphPad Prism 8. *p* < 0.05 was selected as the cut-off to define significance.

## Results

### Changes in lncRNA, miRNA, and mRNA expression following the knockout of miR-147

**FIGURE 1:**
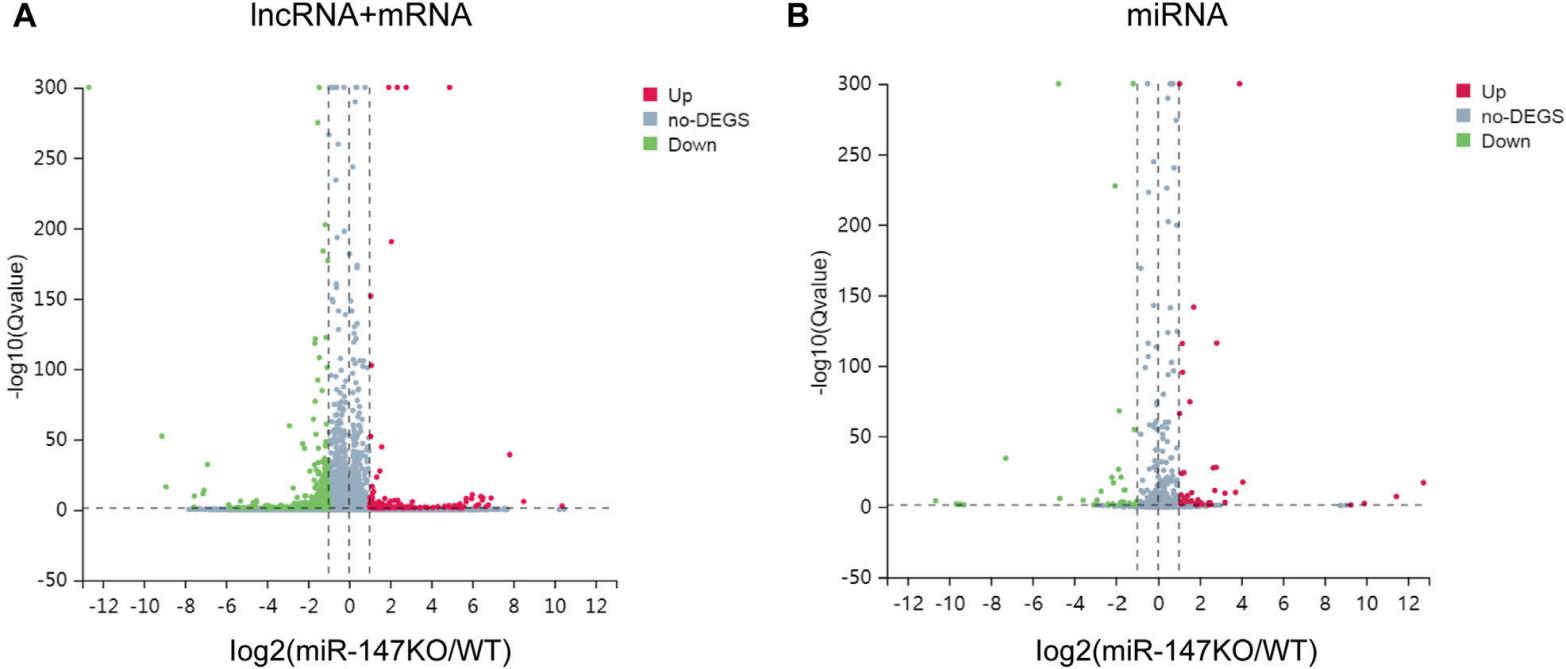
lncRNA, miRNA, and mRNA expression profiles in WT and miR-147^−/−^ mice. **(A)**Volcano plots were generated to identify 91 upregulated and 114 downregulated lncRNAs as well as 71 upregulated and 156 downregulated mRNAs**,** or **(B)** 43 upregulated and 31 downregulated miRNAs. Upregulated and downregulated transcripts are respectively shown in red and green.

To begin exploring changes in transcriptional activity following the loss of miR-147, the lncRNA, miRNA, and mRNA expression profiles of miR-147^−/−^ and wild-type mice were compared. In total, significant up- and downregulation of 71 and 156 mRNAs, respectively, were observed in miR-147^−/−^ mice. Moreover, these mice exhibited 91 and 114 lncRNAs as well as 43 and 31 miRNAs that were significantly upregulated and downregulated, respectively (Q < 0.05, | log2FC | > 1). The most significant up- and downregulation, respectively, was observed in the lncRNAs BGIG10090_53735and Hoxb3os, while the respective miRNAs were mmu-miR-466b-3p (12.75-fold) and mmu-miR-466c-3p (10.65-fold), and mRNAs were Plac9a (8.50-fold) and Ifi30 (12.68-fold). These results were presented using volcano plots (Figure 1), and the top 5 differentially expressed (DE) transcripts in each category are presented in Tables 1–3.

**TABLE 1:** The five lncRNAs showing the most significant up- and downregulation.

Gene ID	Gene symbol	log2 (miRNA-147KO/WT)	Qvalue
BGIG10090_53735	BGIG10090_53735	10.37503943	0.002017084
BGIG10090_66561	BGIG10090_66561	7.820178962	5.96E-40
102632516	Gm30571	6.918863237	4.49E-09
BGIG10090_66134	BGIG10090_66134	6.781359714	3.36E-04
664849	Gm7367	6.491853096	1.06E-07
102634273	Gm31897	−5.87036472	1.89E-04
BGIG10090_65847	BGIG10090_65847	−7.06608919	1.20E-14
BGIG10090_65803	BGIG10090_65803	−7.118941073	3.10E-12
100041965	Oaz1-ps	−7.539158811	1.97E-10
102632302	Hoxb3os	−7.554588852	0.012972808

**TABLE 2:** The five miRNAs showing the most significant up- and downregulation.

Gene ID	log2 (miRNA-147KO/WT)	Qvalue
mmu-miR-466b-3p	12.75279876	7.54E-18
mmu-miR-466o-5p	11.44914865	4.72E-08
mmu-miR-409-5p	9.908392621	0.004727437
mmu-miR-669m-5p	9.257387843	0.045313523
mmu-miR-199b-3p	4.068010479	3.30E-18
mmu-miR-466m-5p	−9.292321633	0.033515257
mmu-miR-147-3p	−9.483815777	0.01829621
novel-mmu-miR351-5p	−9.483815777	0.01829621
novel-mmu-miR456-3p	−9.654636029	0.009939477
mmu-miR-466c-3p	−10.65374078	4.78E-05

**TABLE 3:** The five mRNAs showing the most significant up- and downregulation.

Gene ID	Gene symbol	log2 (miRNA-147KO/WT)	Qvalue
211623	Plac9a	8.499845887	1.32E-06
100041504	LOC100041504	6.672425342	0.006472804
574437	Xlr3b	6.50779464	2.37E-09
102633783	LOC102633783	6.169925001	0.003638533
115490184	Gm42427	6	1.43E-11
545651	Gm13278	−4.906890596	0.012961481
100859931	Gm20604	−6.894817763	5.16E-33
15077	H3c14	−8.915879379	4.48E-17
100039257	Tmem254b	−9.118941073	4.36E-53
65972	Ifi30	−12.67661826	0

### Exploration of the ceRNA regulatory relationships for lncRNAs, miRNAs, and mRNAs modulated by miR-147 knockout

**FIGURE 2:**
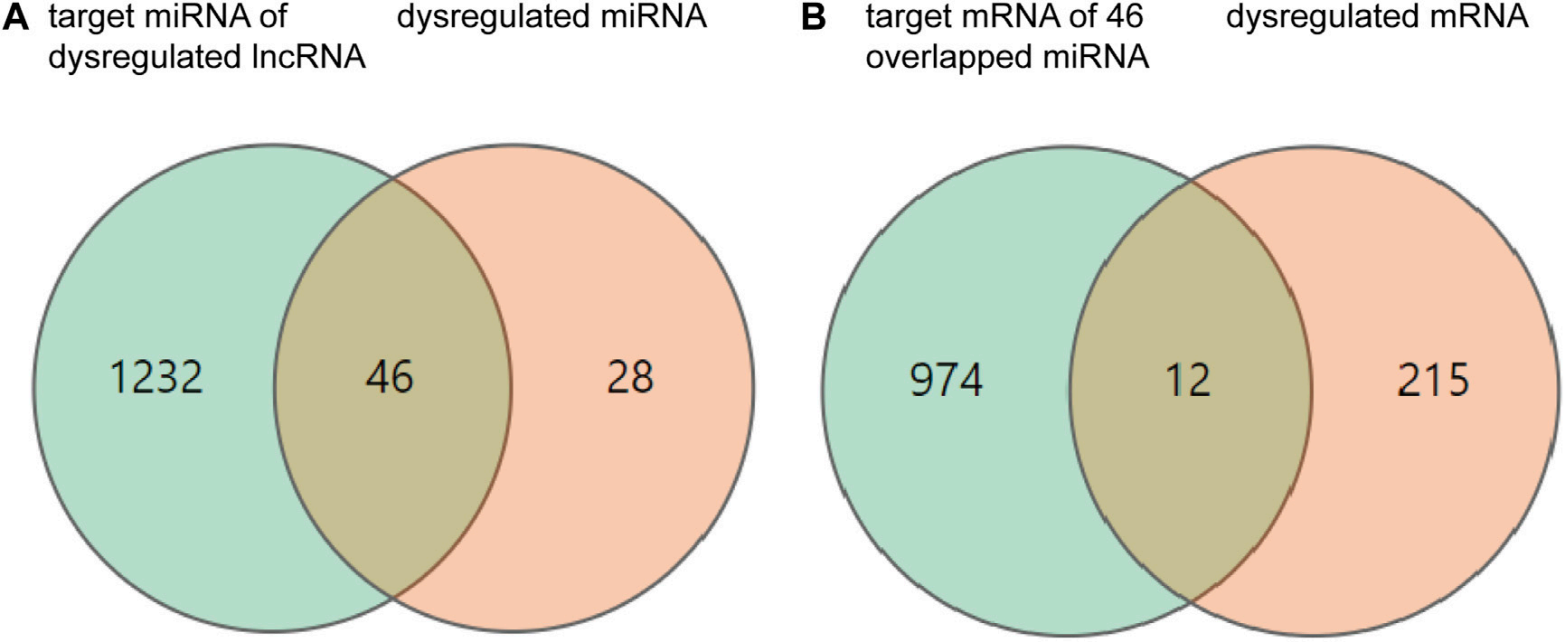
Differentially expressed (DE) mRNAs, miRNAs, and lncRNAs, and their relationships. **(A)** The orange and green circles respectively correspond to 74 DE miRNAs and 1,278 target miRNAs of DE lncRNAs. **(B)** The orange and green circles respectively correspond to 227 DE mRNAs and 986 mRNA targets of DE miRNAs.

**FIGURE 3:**
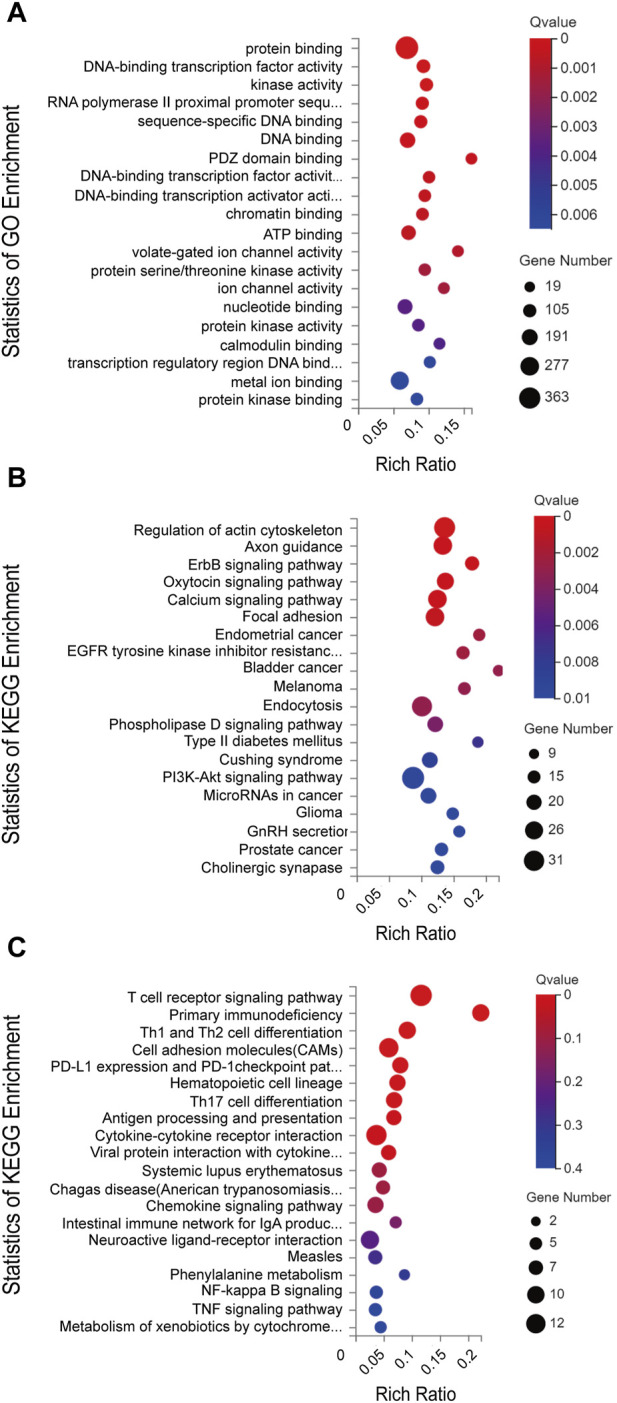
**(A)** GO annotations and **(B)** KEGG pathway analysis results for mRNAs regulated by the established ceRNA network. **(C)** KEGG pathway analysis results for differentially expressed mRNAs. The top 20 results are presented for each analysis based upon Q values.

To explore the possible ceRNA-like regulatory activities of the DE lncRNAs and miRNAs identified in miR-147^−/−^ mice, the putative miRNA targets of DE lncRNAs were identified, and the overlap between this list and DE miRNAs was assessed (Figure 2). In total, 46 overlapping DE miRNAs were identified through this approach, and their putative mRNA targets were then identified and compared with the list of DE mRNAs identified in the above transcriptomic analyses. This approach revealed 12 overlapping DE mRNA target genes. The overlapping target mRNAs of 128 DE lncRNAs and DE miRNAs were also identified (S-Figure 1). GO analyses of 986 putative miRNA target mRNAs were additionally performed, revealing that the most enriched molecular function terms associated with these DE lncRNA and miRNA targets included PDZ domain binding, voltage-gated ion channel activity, and ion channel activity (Figure 3A). GO enrichment analyses were also performed for the cellular component and biological process GO term categories when assessing these mRNA targets (S-Figure 2).

Next, KEGG pathway analyses of mRNA targets of DE miRNAs were conducted, revealing that these genes were enriched in the bladder cancer (Cdh1, Cdkn1a, Dapk2, E2f1, Egfr, Erbb2, Fgfr3, Nras, Mapk1), endometrial cancer (Axin1, Cdh1, Cdkn1a, Egfr, Erbb2, Nras, Pik3cd, Pik3r1, Tcf7l1, Mapk1, Gsk3b), and ErbB signaling (Nrg2, Camk2g, Cdkn1a, Cdkn1b, Egfr, Eif4ebp1, Erbb2, Nras, Pik3cd, Pik3r1, Stat5b, Pak6, Shc2, Mapk1, Gsk3b) pathways (Figure 3B). KEGG enrichment analyses were also performed for DE mRNAs (Figure 3C).

### ceRNA network construction

**FIGURE 4:**
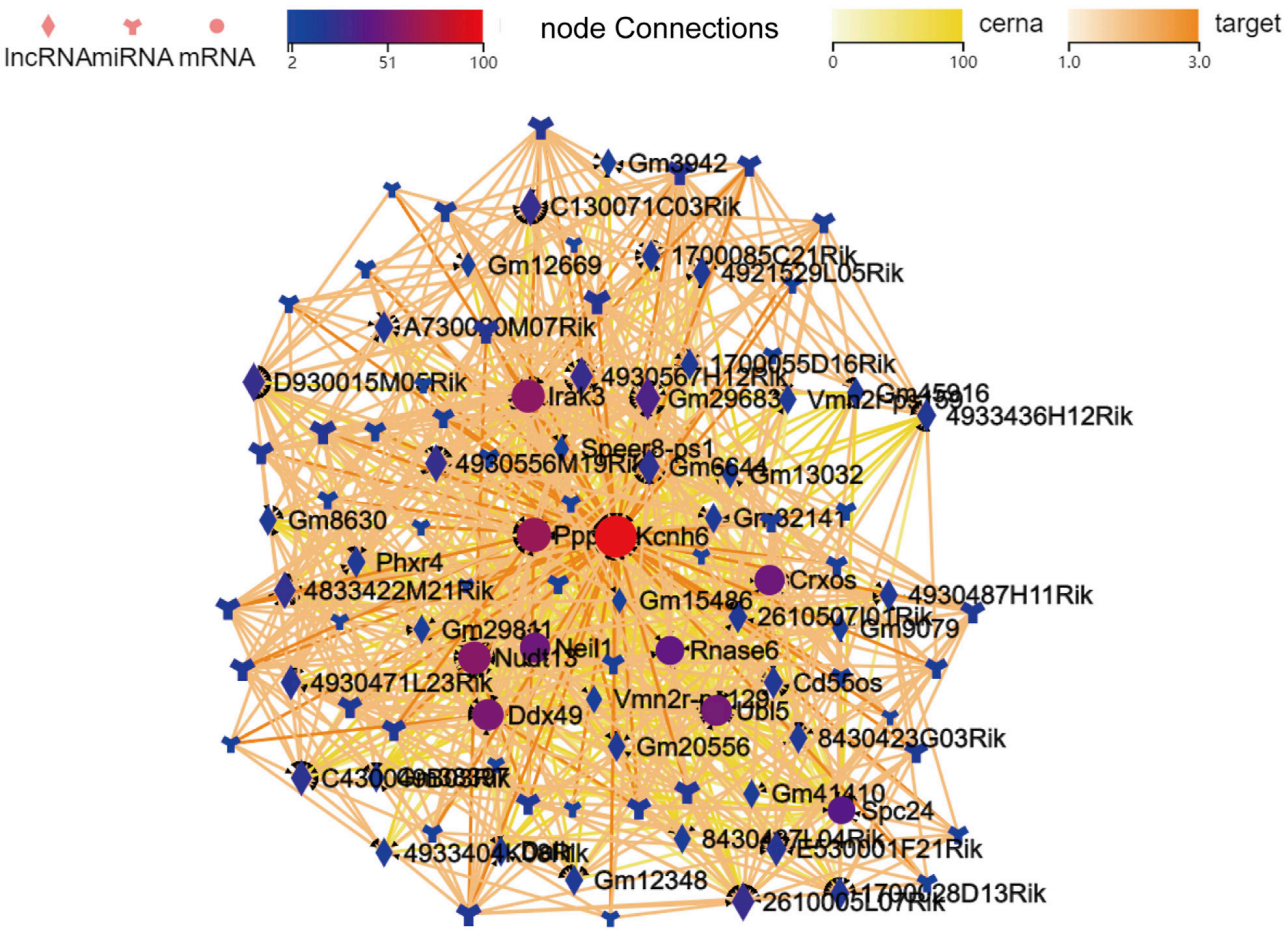
The established lncRNA-miRNA-mRNA network when filtered to focus on voltage-gated ion channel activity-related mRNAs identified in GO analyses. mRNAs, lncRNAs, and branching shapes respectively represent mRNAs, lncRNAs, and miRNAs, with associations between these transcripts being represented by connecting lines between nodes.

To better clarify the significance of the DE lncRNAs and miRNAs identified in miR-147^−/−^ mice, GO analyses were next used to screen for DE mRNAs associated with voltage-gated ion channel activity. Using Kcnh6 as targets, a ceRNA network was then constructed that included 41, 50, and 10 lncRNAs, miRNAs, and mRNAs, respectively (Figure 4). The visualized network constructed based on the selected filtered mRNAs may provide new insight into the mechanistic roles played by miR-147 in cancer. To further clarify the relationships among these different transcripts, the overlapping mRNAs between targets of DE lncRNAs and DE miRNAs were identified and used to establish a ceRNA network (S-Figure 3).

### Kcnh6 is upregulated in miR-147^−/−^ mice

**FIGURE 5:**
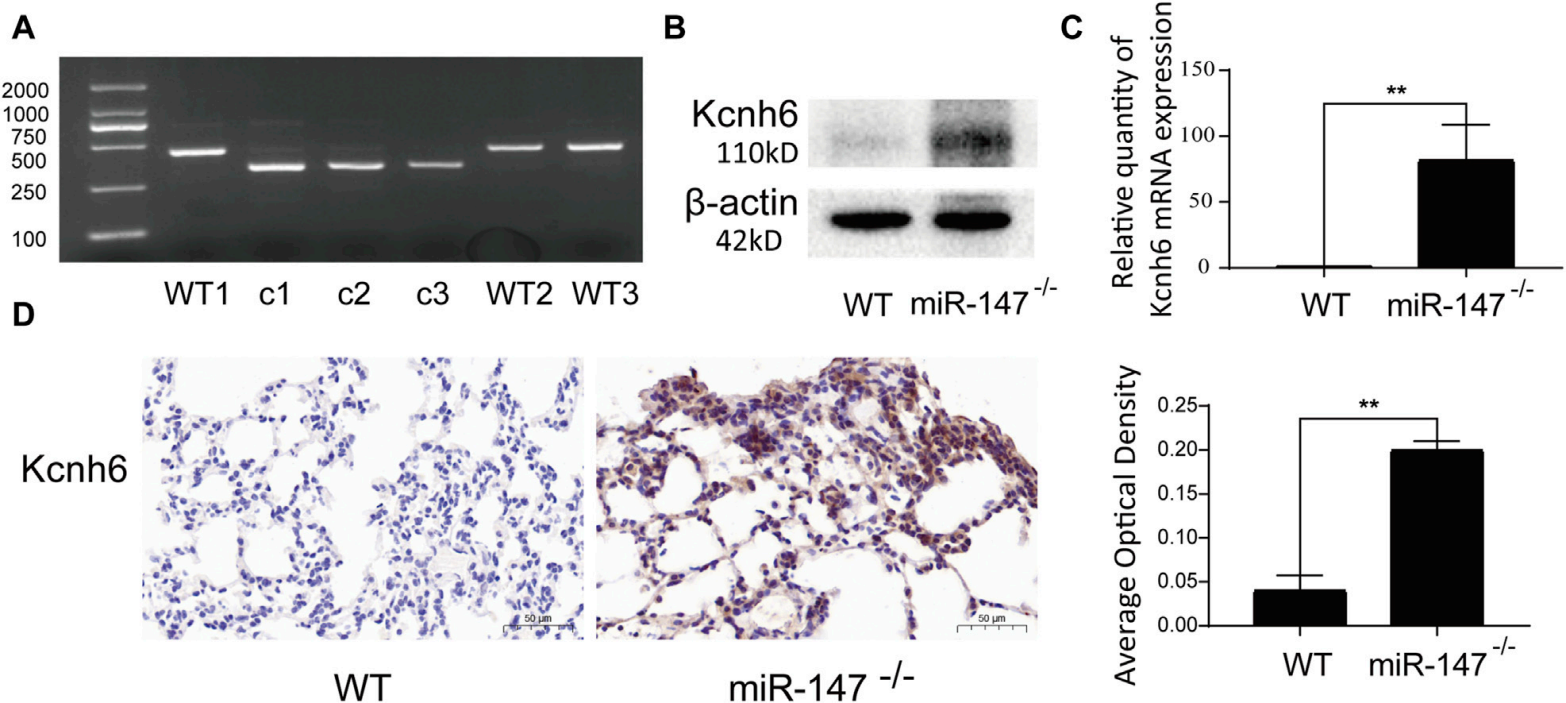
Kcnh6 expression in miR147^−/−^ mice. PCR analyses were used for gene identification. **(A)** In the WT and miR-147^−/−^ mice, the sequences were 435 bp and 324 bp, respectively. Kcnh6 upregulation in miR-147^−/−^ mice was confirmed via **(B)** Western immunoblotting, **(C)** qPCR, and **(D)** immunohistochemistry, with GraphPad Prism 8.0 being used for these analyses. **p < 0.01.

Kcnh6 was identified as a differentially expressed mRNA that was also a core gene within the established lncRNA-miRNA-mRNA network, emphasizing it as being closely related to miR-147. To validate the results of these sequencing analyses, miR-147^−/−^ mice were analyzed, with genotyping confirming that these knockout mice had been successfully established (Figure 5A). WB and qPCR demonstrated significant Kcnh6 upregulation in these mice lacking miR-147 expression (Figures 5B, C), with IHC staining providing additional confirmation of a significant rise in Kcnh6 levels within the lungs of these miR-147^−/−^ mice (Figure 5D). Kcnh6 was also strongly expressed in the livers of the miR-147^−/−^ mice (S-Figure 4).

### Knocking down miR-147 promotes the upregulation of Kcnh6 within embryonic lung fibroblasts

**FIGURE 6:**
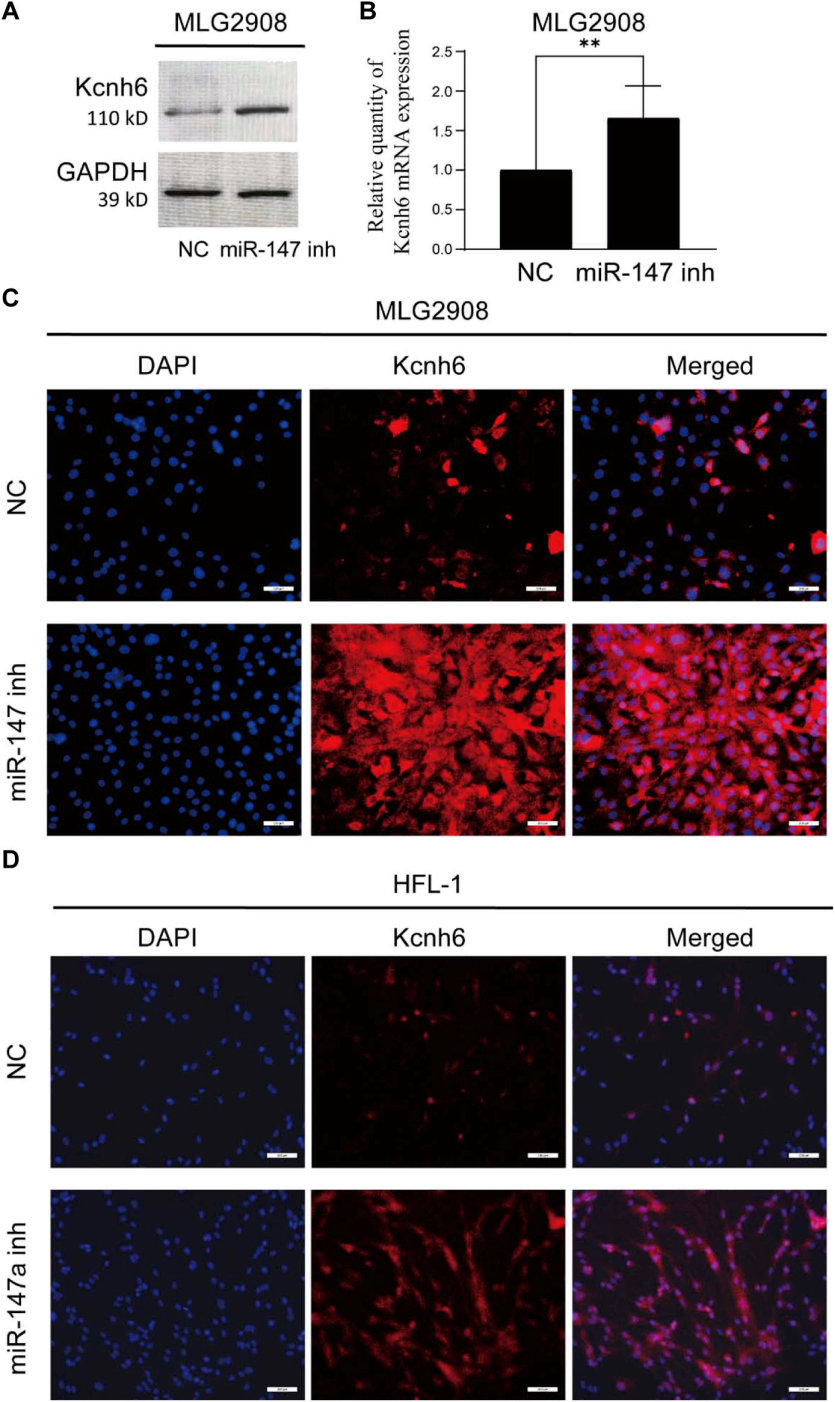
Assessment of the expression of Kcnh6 in miR-147 knockdown cells. After the knockdown of miR-147 within MLG2908 cells, the upregulation of Kcnh6 was detected via **(A)** Western immunoblotting, **(B)** qPCR, and **(C)** immunofluorescence**. (D)** Following miR-147a knockdown within HFL-1 cells, the upregulation of Kcnh6 was detected by immunofluorescence. GraphPad Prism 8.0 was used to analyze the results. **p < 0.01.

To clarify possible modulatory associations between miR-147 and Kcnh6, miR-147 was knocked down in both murine MLG2908 lung fibroblasts and human HFL-1 embryonic lung fibroblasts, after which qPCR, immunofluorescence, and Western immunoblotting were employed to assess Kcnh6 expression. These analyses demonstrated that Kcnh6 protein and mRNA levels rose upon miR-147 knockdown, in line with the results shown above (Figure 6).

## Discussion

Oncogenic transformation occurs through a cumulative process in which multiple incremental changes in cellular characteristics ultimately lead to unrestrained growth. A range of systemic factors, including insulin-related signaling, however, can also induce carcinogenic signaling and shape cancer phenotypes (Dearth et al., 2007). Insulin is secreted into systemic circulation whereupon it binds to cell surface insulin receptors, activating intracellular PI3K signaling activity that may contribute to malignant tumor development (Ruderman et al., 1990; Gallagher and LeRoith, 2011). In line with this hypothesis, hyperinsulinemia is associated with more rapid tumor development in both mice and humans (Novosyadlyy et al., 2010; Orgel and Mittelman, 2013), whereas reducing insulin levels yields the opposite effect (Nencioni et al., 2018). Kcnh6 is a voltage-dependent K^+^ (Kv) channel that primarily influences the secretion of insulin (Vandenberg et al., 2012; Hylten-Cavallius et al., 2017). Indeed, Kcnh6 dysfunction has been reported to result in inappropriate short-term insulin secretion as well as β-cell failure over longer time periods (Yang et al., 2018). The Kv inhibitor BBR has shown promise as an approach to targeting Kcnh6 and thereby treating diabetes in humans (Zhao et al., 2021). Kcnh6 is also capable of reportedly protecting pancreatic β cells against ER-related stress and apoptotic death (Lu et al., 2020). The functional importance of Kcnh6 in tumors, however, remains to be studied in detail.

Several published articles have highlighted the biological functions of miR-147 and suggested that it may offer value as a biomarker for a range of diseases when assessed in combination with other miRNAs or validated biomarkers (Enes et al., 2016; Michalovicz et al., 2019). In one report, HPM was found to alleviate colon damage and suppress related inflammatory activity owing to its ability to promote miR-147 upregulation, thereby reducing inflammation-related mRNA expression and downstream inflammatory cytokine levels (Wei et al., 2015). Other studies have established miR-147 and a lncRNA MEG3 target that is closely tied to the proliferative activity and apoptotic death of leukemia cells *in vitro* (Li et al., 2018). The downregulation of miR-147 has been reported in NSCLC tumors as compared to paracancerous tissues, with corresponding miR-147 downregulation in NSCLC patient serum and further evidence that lower serum miR-147 is independently associated with poorer NSCLC patient prognosis (Chu et al., 2016; Li et al., 2020). In a previous study, our group determined that knocking down miR-147 was associated with significant changes in thymic lncRNA, miRNA, and mRNA expression in mice. In radiation-protected mouse lungs, the ability of troxerutin to upregulate PDPK1 was found to be mediated through miR-147 targeting and the consequent activation of AKT together with the inhibition of JNK activity (Song et al., 2023). To better clarify the role that knocking out miR-147 has on the murine lungs, lung tissues from miR-147^−/−^mice were thus used for sequencing. Here, Kcnh6 upregulation was observed in the lungs of miR-147^−/−^ mice, and associated transcriptomic changes were enriched in tumor-associated signaling pathways. While these results supported the ability of miR-147 to serve as a regulator of Kcnh6, they did not demonstrate the status of Kcnh6 as a direct miR-147 target. Accordingly, the targeting relationship between the two was probed in greater detail. There is not a high degree of miR-147 conservation between mice and humans, with slight differences in the sequences of the mouse (miR-147) and human (miR-147a) versions of this miRNA. To validate the regulatory relationship between miR-147 and Kcnh6, this miRNA was inhibited in both murine and human embryonic lung fibroblasts, yielding comparable results in both cases. This suggests that miR-147 may suppress insulin secretion via the inhibition of Kcnh6, thereby suppressing PI3K signaling activity and influencing tumorigenesis.

Since the first proposal of the ceRNA network model, researchers have increasingly sought to clarify the regulatory links among lncRNAs, mRNAs, and miRNAs based on the hypothesis that MRE-containing ceRNAs can modulate miRNA functionality by competing with target mRNAs for miRNA binding (Salmena et al., 2011). In some reports, lncRNAs have been demonstrated to modulate tumor-associated gene expression through their ability to impact tumor-related mRNA expression (Wang et al., 2020). Here, the overlap between DE miRNAs and the targets of DE lncRNAs was used to identify miRNA-mRNA targeting relationships. This ultimately enabled the construction of a ceRNA network centered on the DEG Kcnh6, providing predictive insight into the lncRNAs, miRNAs, and mRNAs that may be associated with Kcnh6. To date, few articles have focused on tumor treatment via modulating the insulin-PI3K axis, and the present results thus highlight a promising new target for antitumor therapeutic strategies focused on this axis.

## Conclusion

These analyses revealed that abnormal RNA regulation may contribute to the incidence of various malignancies including endometrial, bladder, and lung cancers, with the ErbB signaling playing a key role in the biological changes that develop following the knockout of miR-147 through shifts in the pulmonary lncRNA-miRNA-mRNA network. Further studies of the miR-147-related ceRNA network and efforts to target specific lncRNAs and miRNAs are thus expected to yield new insight into how miR-147 shapes the pathogenesis of cancers and other diseases. However, additional analyses will be essential to validate the lncRNA-miRNA-mRNA network relationships, providing a clear avenue for future research efforts.

## Data Availability

The original contributions presented in the study are included in the article/Supplementary Material, further inquiries can be directed to the corresponding author.
