# Supplementary material for: Evaluation of the lncRNA-miRNA-mRNA ceRNA network in lungs of miR-147 −/− mice
Source: Front Pharmacol. 2024 Mar 6;15:1335374. doi: 10.3389/fphar.2024.1335374 (PMC10953689; doi:10.3389/fphar.2024.1335374)
Supplement: Supplementary file 1 [file Table1.docx]

**SUPPLEMENTARY FIGURES LEGENDS**

**SUPPLEMENTARY FIGURE 1 |** Differentially expressed (DE) mRNAs, miRNAs, and lncRNAs, and relationships among these DE transcripts. The 1734 mRNAs targeted by DE lncRNAs are represented with a green circle, while the 1629 DE mRNAs are represented with a red circle.

**SUPPLEMENTARY FIGURE 2 |** GO enrichment results for mRNAs identified as lncRNA-miRNA-mRNA network regulatory targets. **(A)** GO cellular component enrichment results. **(B)** GO biological process enrichment results.

**SUPPLEMENTARY FIGURE 3 |** Identification of differentially expressed mRNAs targeted by differentially expressed lncRNAs and lncRNA-miRNA-mRNA network establishment.

**SUPPLEMENTARY FIGURE 4 |** Expression of Kcnh6 in the liver of miR-147^-/-^ mice. Immunohistochemistry was used to detect the expression of Kcnh6, and results were analyzed by GraphPad Prism 8.0. *P < 0.05.


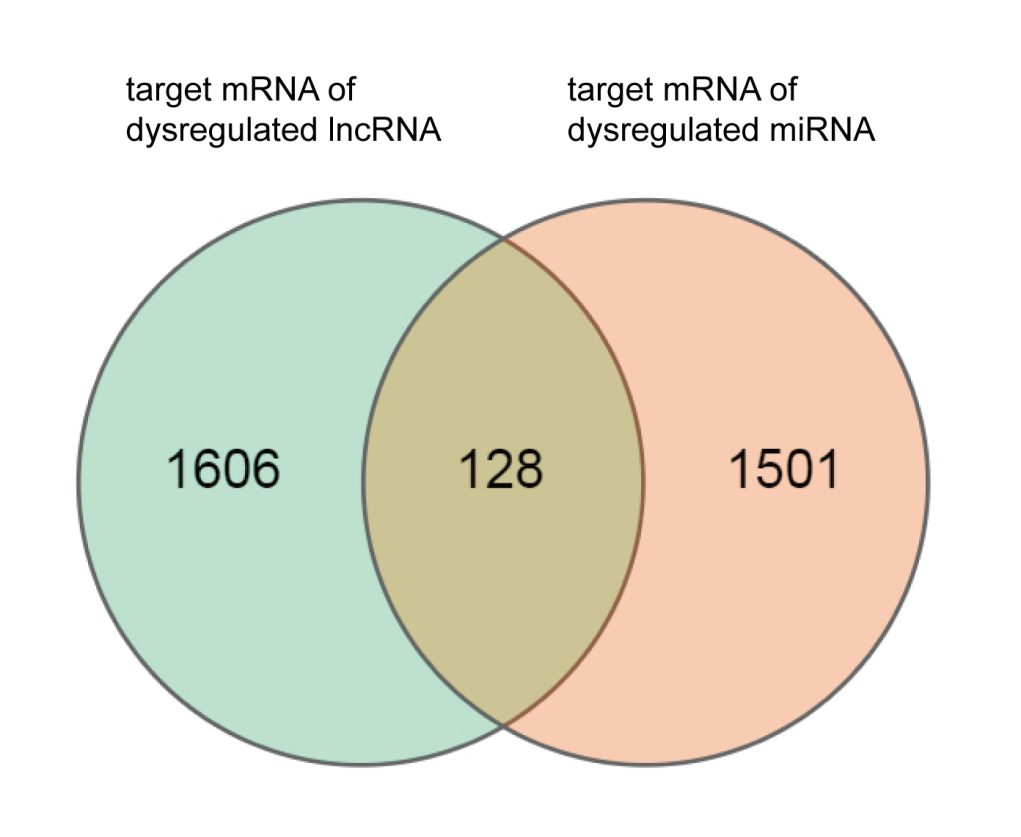


1. Fig. 1


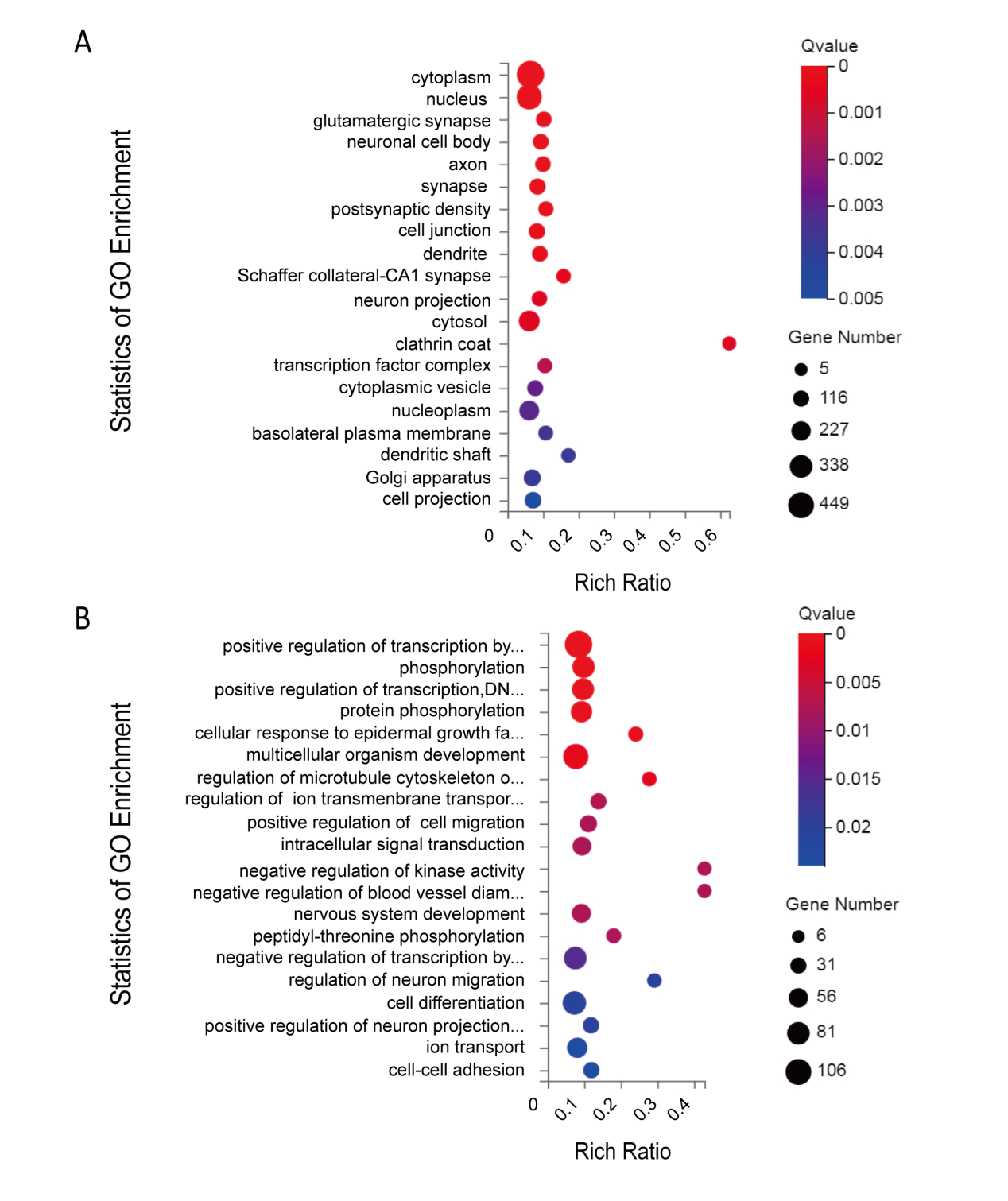


S-Fig. 2


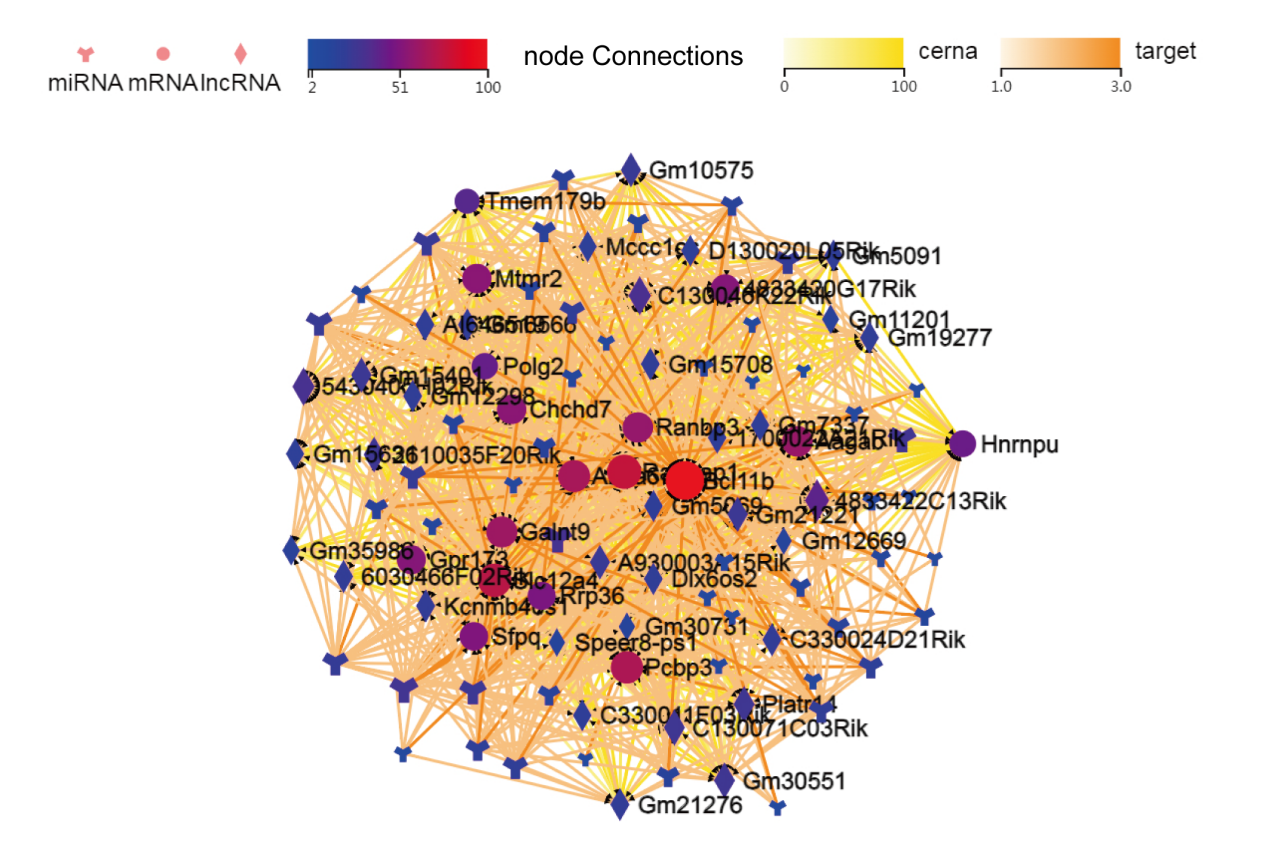


S-Fig. 3


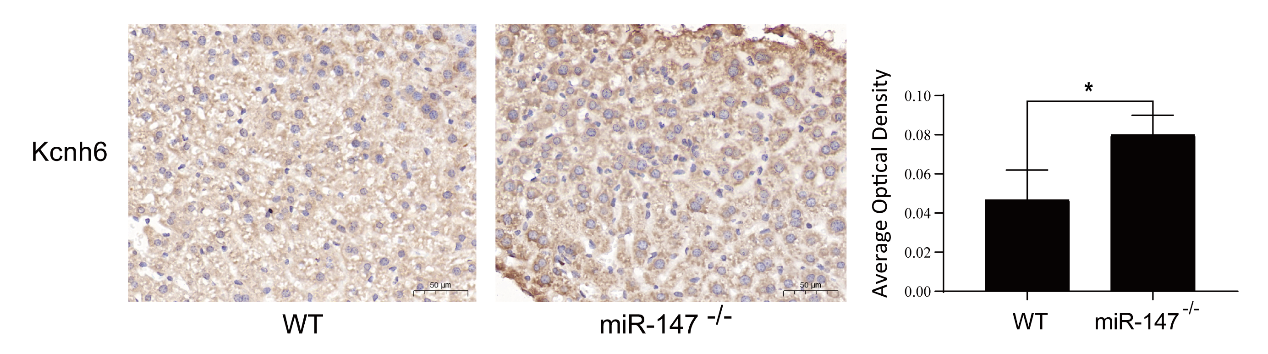
S-Fig. 4
