# Supplementary figures and images for: Evaluation of the lncRNA-miRNA-mRNA ceRNA network in lungs of miR-147 −/− mice
Source: Front Pharmacol. 2024 Mar 6;15:1335374. doi: 10.3389/fphar.2024.1335374 (PMC10953689; doi:10.3389/fphar.2024.1335374)

**Original Data**


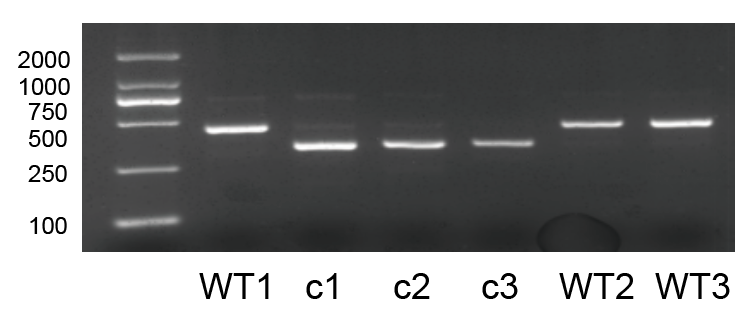


Fig. 5A


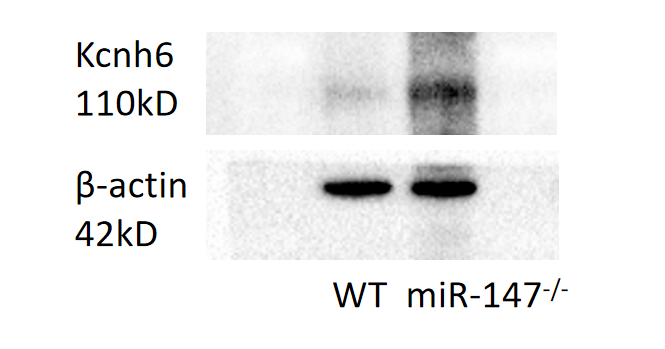


Fig. 5B


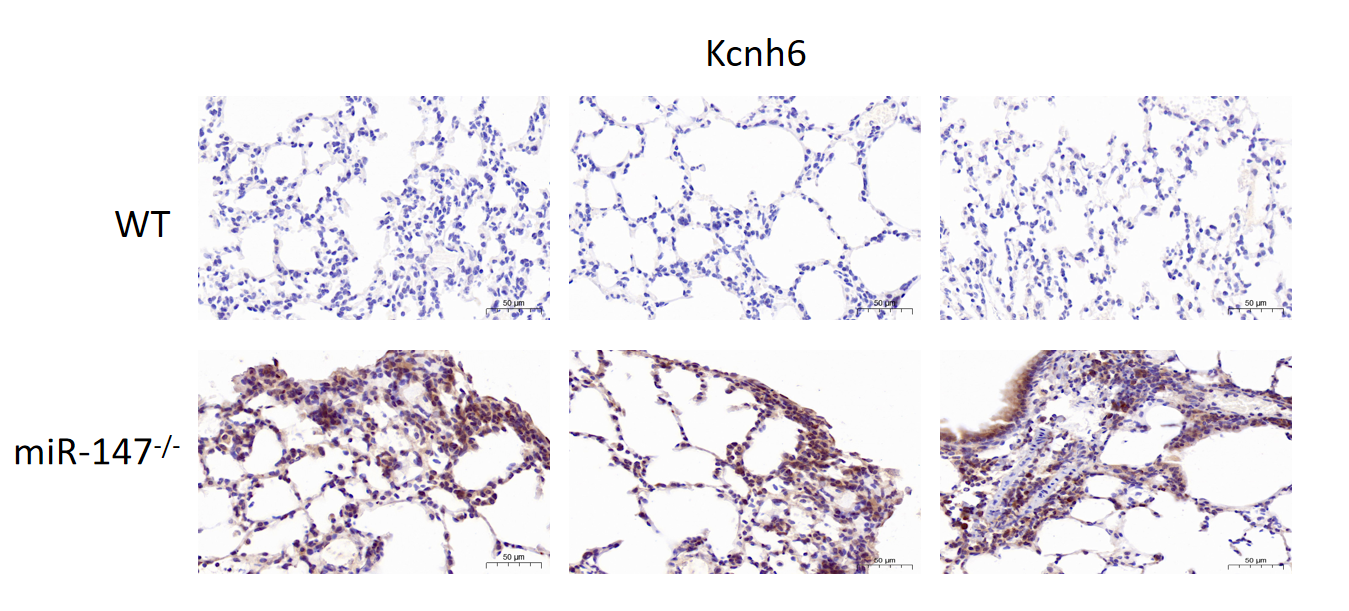


Fig. 5D


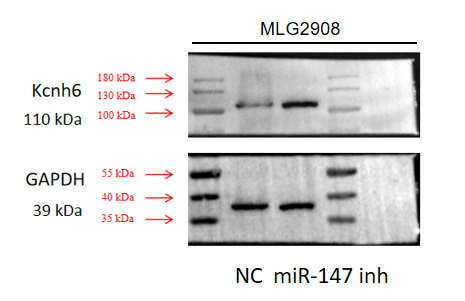


Fig. 6A


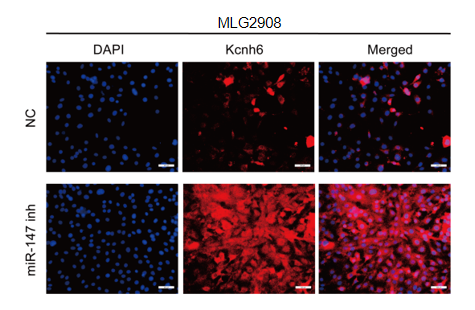


Fig. 6C


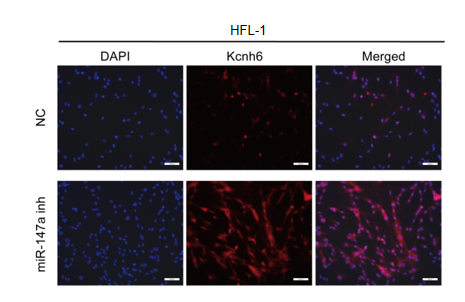


Fig. 6D


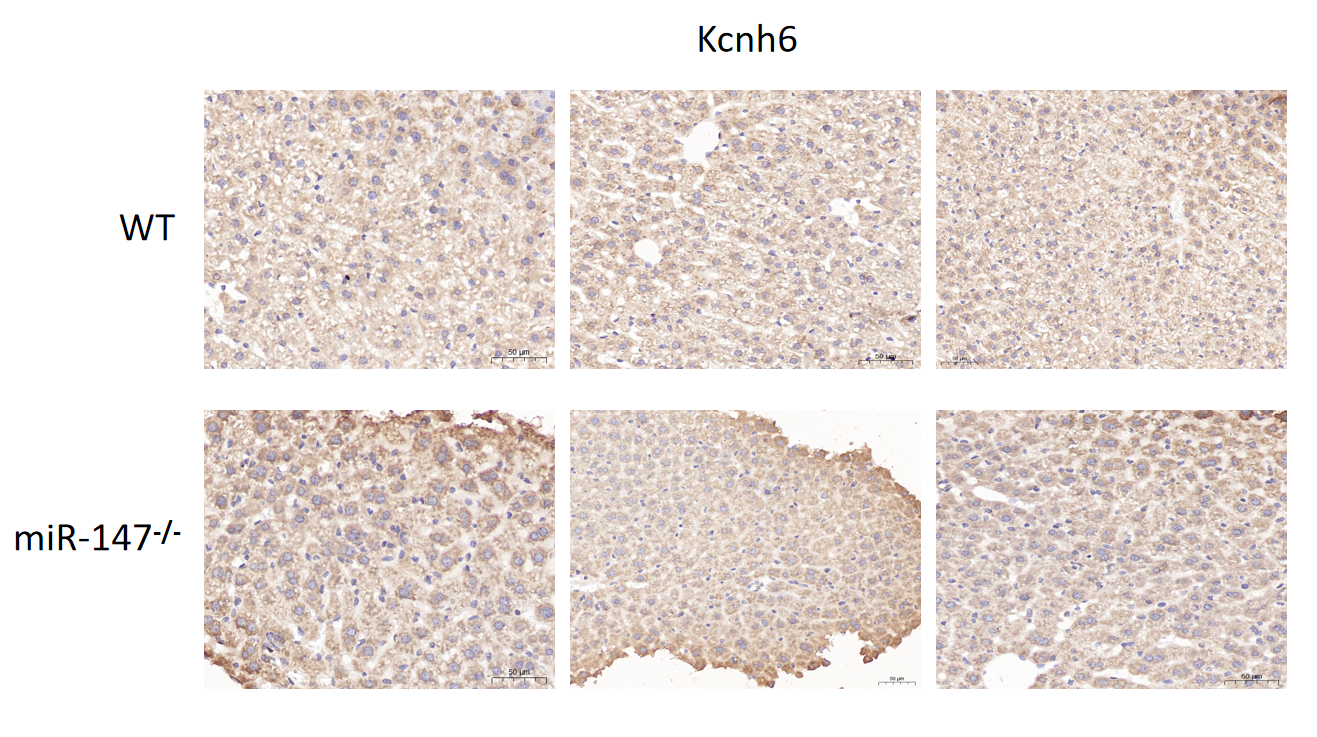


S-Fig. 4

Supplement: Supplementary file 2 [file DataSheet1.zip › Original Data/Original Data.docx]
